# Infection with SARS-CoV-2 Is Associated with Elevated Levels of IP-10, MCP-1, and IL-13 in Sepsis Patients

**DOI:** 10.3390/diagnostics13061069

**Published:** 2023-03-11

**Authors:** Tanja Eichhorn, Silke Huber, René Weiss, Marie Ebeyer-Masotta, Lucia Lauková, Robert Emprechtinger, Rosa Bellmann-Weiler, Ingo Lorenz, Judith Martini, Markus Pirklbauer, Dorothea Orth-Höller, Reinhard Würzner, Viktoria Weber

**Affiliations:** 1 Department for Biomedical Research, University for Continuing Education Krems, 3500 Krems, Austria; tanja.eichhorn@donau-uni.ac.at (T.E.); rene.weiss@donau-uni.ac.at (R.W.); marie.ebeyer-masotta@donau-uni.ac.at (M.E.-M.); lucia.krajcik-laukova@donau-uni.ac.at (L.L.); 2 Institute of Hygiene and Medical Microbiology, Medical University of Innsbruck, 6020 Innsbruck, Austria; silke.huber@i-med.ac.at (S.H.); reinhard.wuerzner@i-med.ac.at (R.W.); 3 Faculty of Health and Medicine, University for Continuing Education Krems, 3500 Krems, Austria; robert.emprechtinger@donau-uni.ac.at; 4 Department of Internal Medicine II, Medical University of Innsbruck, 6020 Innsbruck, Austria; rosa.bellmann-weiler@i-med.ac.at; 5 Department of General and Surgical Critical Care Medicine, Medical University of Innsbruck, 6020 Innsbruck, Austria; ingo.lorenz@i-med.ac.at; 6 Department of Anaesthesia and Intensive Care Medicine, Medical University of Innsbruck, 6020 Innsbruck, Austria; judith.martini@i-med.ac.at; 7 Department of Internal Medicine IV-Nephrology and Hypertension, Medical University Innsbruck, 6020 Innsbruck, Austria; markus.pirklbauer@i-med.ac.at; 8 MB-LAB Clinical Microbiology Laboratory, 6020 Innsbruck, Austria; do@mb-lab.com

**Keywords:** sepsis, COVID-19, immunothrombosis, extracellular vesicles, inflammation

## Abstract

Immunothrombosis, an excessive inflammatory response with simultaneous overactivation of the coagulation system, is a central pathomechanism in sepsis and COVID-19. It is associated with cellular activation, vascular damage, and microvascular thrombosis, which can lead to multiple organ failure and death. Here, we characterized factors related to immunothrombosis in plasma samples from 78 sepsis patients. In the course of routine clinical testing, SARS-CoV-2 was detected in 14 of these patients. Viral infection was associated with a higher mortality. Both, COVID-19 negative and COVID-19 positive sepsis patients showed increased levels of effectors of immunothrombosis, including platelet factor 4, D-dimer, nucleosomes, citrullinated histone H3, high mobility group box-1 protein, as well as phosphatidylserine-expressing platelet-derived extracellular vesicles, compared to healthy controls (*n* = 25). Using a 27-plex cytokine bead array, we found that Interleukin (IL)-1ra, IL-6, IL-8, IL-13, tumor necrosis factor (TNF)-α, interferon inducible protein (IP)-10, monocyte chemotactic protein (MCP)-1, macrophage inflammatory protein (MIP)-1α, and granulocyte-colony stimulating factor (G-CSF) were elevated in both, COVID-19 negative and COVID-19 positive sepsis patients, as compared to healthy controls. SARS-CoV-2 infection was associated with elevated levels of IP-10, MCP-1, and IL-13, while all other mediators widely overlapped between COVID-19 negative and COVID-19 positive patients.

## 1. Introduction

Sepsis is a life-threatening systemic inflammatory syndrome triggered by an inadequate host response to infection [1]. COVID-19, caused by the severe acute respiratory syndrome coronavirus SARS-CoV-2, shares many pathophysiological and clinical features with sepsis, including coagulopathy and vascular microthrombosis, as well as multiple organ failure [2,3,4], which accounts for most of the deaths in COVID-19 [5].

The thrombotic events associated with sepsis are best described as immunothrombosis, a simultaneous overactivation of the innate immune system and coagulation [6,7,8]. Dysregulated complement activation enhances neutrophil activation and recruitment to the lungs, and induces tissue factor (TF) expression on neutrophils, monocytes, and endothelial cells, resulting in microvascular thrombosis and endothelial dysfunction [2]. The release of neutrophil extracellular traps (NETs), decondensed DNA associated with neutrophil elastase, antimicrobial peptides, histones, and high mobility group box-1 protein (HMGB-1) further promotes thrombosis and tissue damage [9]. NETs trigger coagulation via both, the intrinsic (FXII activation) [10] and the extrinsic (TF exposure) pathways [11].

Next to neutrophils, platelets are the main effectors of immunothrombosis [12,13,14]. Platelet activation triggers the release of platelet factor 4 (PF4, CXCL4), supporting monocyte and neutrophil recruitment [15]. Activated platelets secrete HMGB-1 and promote NETosis [16,17], and enhanced aggregate formation of activated platelets with monocytes and neutrophils has been linked to thrombotic complications in severe COVID-19 [9,18,19,20]. Activated platelets are a major source of circulating extracellular vesicles (EVs) [21,22], which expose phosphatidylserine and serve as a catalytic surface for thrombin generation [23,24].

Here, we analyzed a panel of 47 parameters related to immunothrombosis in sepsis patients and found that patients with confirmed SARS-CoV-2 infection had higher levels of IP-10, MCP-1, and IL-13.

## 2. Materials and Methods

### 2.1. Patients and Sample Collection

Between October 2019 and July 2021, 78 sepsis patients with suspected bloodstream infection and a sequential organ failure assessment (SOFA) score > 2 hospitalized at the Department of Internal Medicine or the Department of Operative Medicine, both Medical University of Innsbruck, Austria, were enrolled in this study. Collection of EDTA-anticoagulated whole blood using an aspiration-based blood collection system (S-Monovette^®^, Sarstedt, Goetzis, Austria) was performed during routine venipuncture for blood culture testing. Whole blood was centrifuged (2000× *g*, 15 min, 22 °C) within 4 h to obtain platelet-poor plasma and stored in aliquots at −80 °C until further analysis. Of the 78 sepsis patients, 14 were additionally diagnosed with COVID-19 using the cobas^®^ SARS-CoV-2 Qualitative RT-PCR Test (Roche, Mannheim, Germany) for the detection of the target *E gene* (pan-Sarbecovirus) and the SARS-CoV-2 specific target *orf1a/b gene*. The control group (*n* = 25) consisted of healthy individuals from whom EDTA-anticoagulated blood was collected after written consent and directly processed.

### 2.2. Data Collection

Clinical data collected from the electronic medical record included the date of sampling, gender, age, ward, the result of routine blood culture (BC), clinical diagnosis, death within one month after sampling, antimicrobial treatment, puncture site, C-reactive protein (CRP), procalcitonin (PCT), red blood cell, white blood cell, monocyte, neutrophil, lymphocyte, and platelet counts, as well as neutrophil-to-lymphocyte ratio (NLR). The 14 COVID-19 patients were classified according to the WHO severity criteria (moderate, *n* = 6; severe, *n* = 5; critical, *n* = 3) [25].

### 2.3. Blood Culture

Routine blood culture testing was performed on the wards by inoculating at least one set of BACTEC™ Plus Aerobic/F and BACTEC™ Lytic/10 Anaerobic/F culture bottles (both BD Diagnostics, Vienna, Austria) with the patient’s whole blood. Culture bottles were immediately transported to the routine microbiological laboratory and incubated at 37 °C using the BACTEC FX Packaged continuous BC monitoring system (BD Diagnostics) until growth or for 5 days post-inoculation. Positive BCs were further used for pathogen identification after cultivation on agar plates using matrix-assisted laser desorption/ionization time of flight mass spectrometry (MALDI-TOF MS) based on the reference Biotyper library v4.1 (both Bruker Daltonik, Bremen, Germany) and antimicrobial susceptibility testing (AST). Disk diffusion was used for the latter and was performed in accordance with the current EUCAST guidelines at the time point of sampling [26].

### 2.4. Coagulation-Related Parameters

In addition to platelet counts, plasma concentrations of PF4 and D-dimer were quantified by ELISA (R&D Systems, Minneapolis, MN, and Technozym, Vienna, Austria, respectively). The cell death detection ELISA (Roche, Mannheim, Germany) was used to quantify nucleosomes in plasma. Results are indicated as arbitrary units (AU), which represent the ratio of the maximum measured absorbance and the absorbance of the individual samples. Citrullinated histone H3 and HMGB-1 were quantified by ELISA (Cayman Chemical, Ann Arbor, MI, and IBL International, Hamburg, Germany).

### 2.5. Characterization of Extracellular Vesicles

EVs were characterized using a CytoFLEX LX flow cytometer (Beckman Coulter, Brea, CA, USA) equipped with 405 nm, 488 nm, 561 nm, and 631 nm lasers. For staining, samples were diluted 1:100 in sterile-filtered Annexin V (Anx5) binding buffer (0.1 μm Minisart syringe filter, Sartorius Stedim Biotech, Goettingen, Germany). Aliquots (100 μL) of the diluted samples were incubated for 15 min at room temperature in the dark with APC-conjugated Anx5 (BD Biosciences, San Jose, CA, USA) as a marker for EVs exposing phosphatidylserine in combination with PE-conjugated anti-hCXCL4 (R&D Systems) and PC7-conjugated anti-CD41 or with FITC-conjugated anti-CRP (Abcam, Cambridge, UK). All fluorochrome conjugates and the respective antibody clones are listed in Supplementary Table S1. To remove potential precipitates, fluorochrome conjugates were centrifuged at 18,600× *g* for 10 min at 4 °C prior to use. Stained samples were diluted 1:5 in sterile-filtered Anx5 binding buffer (BD Biosciences) prior to analysis. Calibration of the flow cytometer was performed with fluorescent silica beads (1 μm, 0.5 μm, 0.1 μm; excitation/emission 485/510 nm; Kisker Biotech, Steinfurt, Germany). The triggering signal for EVs was set to the violet side scatter (405 nm), and the EV gate was set below the 1 µm bead cloud (Supplementary Figure S1) as previously described [27,28]. Acquisition was performed for 2 min at a flow rate of 10 µL/min. Data were analyzed using the Kaluza Software (Beckman Coulter, version 2.1). EVs were identified as Anx5^+^ events in the EV gate.

### 2.6. Quantification of C5b-9

Levels of the C5b-9 (terminal complement complex, TCC) were measured using an in-house sandwich ELISA as previously described [29]. Briefly, 96-well medium-binding microplates (Greiner Bio-One, St. Gallen, Switzerland) were coated with a monoclonal anti-human C9 neoantigen antibody (Hycult Biotech, Uden, The Netherlands). Following sample incubation, a custom-made biotinylated polyclonal anti-human C7 antibody [30] and avidin-alkaline phosphatase (Millipore, Sigma, Merck KGaA, Darmstadt, Germany) were used to detect bound C5b-9. Zymosan-activated human serum was used as standard.

### 2.7. Quantification of Cytokines, Chemokines, and Growth Factors

A set of 27 cytokines, chemokines, and growth factors were analyzed by bead array (Bio-Plex Pro human cytokine 27-plex; Bio-Rad, Hercules, CA, USA), including interleukin (IL)-1β, IL-1 receptor antagonist (IL-1ra), IL-2, IL-4, IL-5, IL-6, IL-7, IL-8, IL-9, IL-10, IL-12p70, IL-13, IL-15, IL-17A, interferon-gamma (IFN-γ), tumor necrosis factor-alpha (TNF-α), eosinophil chemotactic protein (eotaxin), interferon-gamma inducible protein-10 (IP-10), monocyte chemotactic protein-1 (MCP-1), macrophage inflammatory protein-1 alpha and beta (MIP-1α, MIP-1β), regulated on activation, normal T-cell expressed and secreted (RANTES), basic fibroblast growth factor (FGF), granulocyte colony-stimulating factor (G-CSF), granulocyte-macrophage colony-stimulating factor (GM-CSF), platelet-derived growth factor (PDGF), as well as vascular endothelial growth factor (VEGF). Plasma samples were diluted 1:4 with sample diluent and analyzed according to the instructions of the manufacturer using the Bio-Plex 200 System and the Bio-Plex Manager software version 5.0 (Bio-Rad).

### 2.8. Statistical Analysis

Statistical analysis was carried out using R (version 4.1.3). We reported the bias-corrected and accelerated (BCa) bootstrap 95% confidence intervals (CI) of the median. If the bootstrapping confidence interval returned the warning “extreme order statistics used as endpoints”, we used the percentage bootstrap confidence interval. Calculation of confidence intervals was performed by nonparametric bootstrapping using the boot package with 100,000 iterations [31,32]. Correlation analysis was performed using the Spearman correlation test and heat map analyses of the correlation coefficients were performed.

## 3. Results

### 3.1. Patient Characteristics

**Figure 1:**
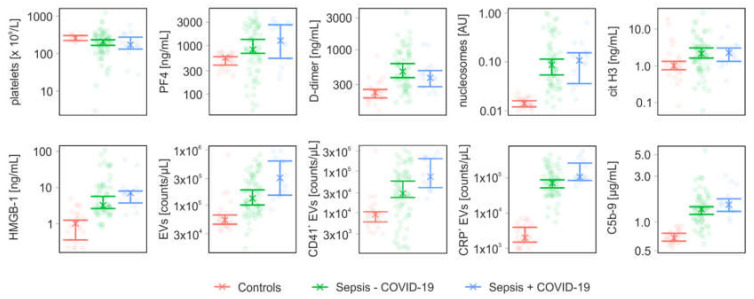
Levels of coagulation- and complement-related parameters in sepsis patients (Sepsis − COVID-19), sepsis patients diagnosed with COVID-19 (Sepsis + COVID-19), and healthy controls. The x represents the median of the measurements. (PF4, platelet factor 4; cit H3, citrullinated histone H3; HMGB-1, high mobility group box-1 protein; EVs, extracellular vesicles; CRP, C-reactive protein; C5b-9, terminal complement complex).

**Figure 2:**
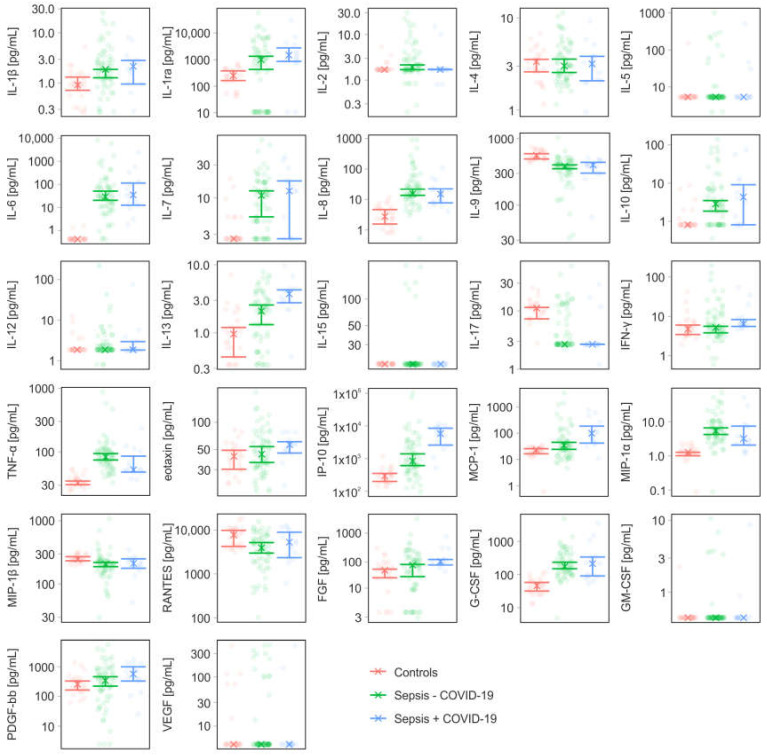
Profiles of cytokines, chemokines, and growth factors in sepsis patients without (Sepsis − COVID-19) and with COVID-19 (Sepsis + COVID-19), and healthy controls. The x represents the median of the measurements.

Samples from 78 sepsis patients were analyzed in this study. Fourteen of these patients were diagnosed with COVID-19 during routine clinical testing in the course of the pandemic, with mean Ct values of 27.9 for the orf1a/b gene and 28.3 for the E gene. Twenty-five healthy individuals served as controls. The patient characteristics are summarized in Table 1. The in-hospital mortality was 11% for COVID-19 negative and 43% for COVID-19 positive patients. Bacteremia was confirmed by blood culture in 9 of the 78 sepsis patients (COVID-19 negative, *n* = 8; COVID-19 positive, *n* = 1). The panel of identified pathogens included *Staphylococcus aureus* (*n* = 4), *Pseudomonas aeruginosa* (*n* = 2), *Escherichia coli* (*n* = 2), *Klebsiella pneumoniae* (*n* = 1), *Streptococcus pneumoniae* (*n* = 1), *Enterobacter cloacae* (*n* = 1), and *Staphylococcus epidermidis* (*n* = 1). Hereby, two patients suffered from co-infections with up to three different pathogens, and *Pseudomonas aeruginosa* was isolated from one COVID-19 positive patient. Blood culture-positive patients exhibited higher PCT levels than blood culture-negative patients, 16.78 (CI 1.61–71.46) µg/L vs. 0.42 (CI 0.2–0.54) µg/L, respectively. The profiles of inflammatory mediators for COVID-19 positive and negative patients are summarized in Table 2. The parameters presented in Table 2 were grouped into factors related to coagulation and complement activation (Figure 1), as well as cytokines, chemokines, and growth factors (Figure 2). Mediator profiles of blood culture-negative and blood culture-positive patients are summarized in Supplementary Table S2, and mediator profiles of survivors and non-survivors are compiled in Supplementary Table S3. Spearman correlation coefficients for all parameters are summarized in Supplementary Figures S2 and S3.

### 3.2. Coagulation- and Complement-Related Parameters

Coagulation- and complement-related parameters included platelet counts, PF4, D-dimer, nucleosomes, citrullinated histone H3, HMGB-1, EV counts, as well as the terminal complement complex C5b-9. EVs were further analyzed regarding their platelet origin (CD41^+^) and their association with CRP (CRP^+^). Both patient groups showed a trend towards lower platelet counts, however, the confidence intervals for both groups overlapped with the control group. D-dimer was elevated in both patient groups. PF4 was increased in COVID-19 negative patients, while the confidence intervals overlapped with the control for COVID-19 positive patients.

We also measured markers of NET formation and observed increased nucleosome and citrullinated histone H3 levels in both patient groups. Likewise, HMGB-1, a known inducer of NET formation, as well as C5b-9 were elevated in both patient groups.

EVs shed from the plasma membrane expose phosphatidylserine and thereby provide a catalytic surface mediating thrombin generation [23,24]. Both, total EV counts and platelet-derived EVs were elevated in COVID-19 positive and COVID-19 negative patients (Figure 1). Both groups showed elevated levels of CRP^+^ EVs, with 55% (CI 46–61%) and 62% (CI 40–68%) CRP^+^ EVs in COVID-19 negative and COVID-19 positive patients, respectively, confirming previous results [33], while only 4% (CI 2–4%) of all EVs were associated with CRP in healthy controls. Percentages of CRP^+^ EVs correlated with CRP levels in both patient groups (Supplementary Figures S2 and S3).

### 3.3. Cytokines, Chemokines, and Growth Factors

COVID-19 positive and COVID-19 negative patients had increased levels of IL-1ra, IL-6, IL-8, IL-13, TNF-α, IP-10, MCP-1, MIP-1α, and G-CSF (Figure 2). FGF was elevated in COVID-19 positive, but not in COVID-19 negative sepsis patients. IP-10, MCP-1, and IL-13 were higher for COVID-19 positive than for COVID-19 negative patients. Both patient groups exhibited lower levels of IL-9 and IL-17 as compared to healthy donors. Mediator levels were not different for survivors and non-survivors (Supplementary Table S3).

## 4. Discussion

Blood culture confirmed bacterial infection in 11.5% of the 78 sepsis patients included in our study (13% of the COVID-19 negative and 7% of the COVID-19 positive sepsis patients). Viral co-infection was associated with a higher prevalence of ICU admission and a higher mortality. Our data confirm recent meta-analyses reporting bacterial co-infections in 7–8% of COVID-19 patients, most frequently in those with critical illness [34,35]. The close connection between bacterial and viral infection is highlighted by recent work on mechanisms leading to gut dysbiosis in COVID-19, showing that SARS-CoV-2 can induce dysbiosis by triggering intestinal inflammation, by dysregulating the angiotensin-converting enzyme 2 (ACE2), which is essential for amino acid metabolism, and by infection of intestinal bacteria [36,37].

The dysregulated immune response in COVID-19 is associated with an enhanced release of inflammatory mediators [38,39,40,41,42,43]. Several studies, however, have challenged the concept of a cytokine storm that would differentiate COVID-19 from sepsis [44,45,46,47]. In line with previous studies, our data suggest that inflammatory mediator profiles in sepsis patients who tested positive for SARS-CoV-2 do not substantially differ from those of critically ill patients suffering from sepsis or acute respiratory distress syndrome [48]. We found increased levels of IL-1ra, IL-6, IL-8, IL-13, TNF-α, IP-10, MCP-1, MIP-1α, and G-CSF in both, COVID-19 negative and COVID-19 positive sepsis patients, as compared to healthy controls. Three out of the 27 factors included in the cytokine bead array, IP-10, MCP-1, and IL-13, were higher in COVID-19 positive than in COVID-19 negative sepsis patients. The chemokine IP-10 is mainly released by lymphocytes, neutrophils, and endothelial cells in response to IFNs and lipopolysaccharide to mediate the recruitment of immune cells [49]. Elevated IP-10 levels, which increased with disease progression, have been described in sepsis patients [50]. Elevation of IP-10 was also observed in COVID-19, where several studies demonstrated an association of IP-10 with increased viral titers and disease severity [39,51]. Along with IP-10, MCP-1 has been associated with disease progression in COVID-19 patients in previous studies, as well [39,52,53]. Consistent with our findings, Donlan and co-workers detected significantly increased IL-13 levels in COVID-19 patients [54]. IL-13 is a type-2 cytokine for the recruitment of eosinophils and M2 macrophages to the lung and is implicated in smooth muscle cell proliferation as well as in mucus production and fibrosis [55]. Its elevation has been closely related to respiratory failure requiring mechanical ventilation, while its blockade showed the potential to ameliorate disease severity [54].

The inflammatory responses in sepsis and in COVID-19 are highly dynamic, resulting in pronounced heterogeneity of patient cohorts. Under this aspect, it is essential to consider that most studies, including ours, are limited to the analysis of single time points rather than providing time courses. Therefore, they can only yield snapshots of the dynamic inflammatory response. From this point of view, a recent prospective study by Loftus and co-workers is particularly valuable. They compared inflammatory and immunosuppressive profiles of 30 critically ill patients with SARS-CoV-2 infection with ten critically ill bacterial sepsis patients at five-time points over a period of 21 days. According to this study, bacterial sepsis was rather associated with early, severe inflammation and profound immunosuppression, while SARS-CoV-2 patients had less severe early inflammation, but persistent immunosuppression, making them particularly vulnerable to secondary infection [56].

The activation and interaction of platelets, neutrophils, and monocytes are major drivers of immunothrombosis. Elevated levels of PF4 have been linked to enhanced platelet activation [57,58,59] and to a hypercoagulable state associated with increased D-dimer levels in sepsis and COVID-19 [9,18,60]. Our data support these observations, since both, COVID-19 negative and COVID-19 positive sepsis patients had increased PF4 and D-dimer levels compared to healthy individuals. However, we could not confirm previous findings [46,61] on distinct D-dimer levels among sepsis and COVID-19 patients.

Activated neutrophils exacerbate immunothrombosis by releasing NETs, DNA fibers decorated with antimicrobial peptides and histones. Exaggerated NET formation has been correlated with disease severity and poor clinical outcome in sepsis as well as COVID-19 [9,62,63,64,65]. NETosis is induced by a range of proinflammatory mediators, including IL-1β and IL-8, while the increased generation of reactive oxygen species is a crucial intracellular process triggering NETosis. Platelet activation represents another route of NET generation, and platelets enhance NETosis by interacting with neutrophils through TLR4, PF4, and extracellular vesicle-dependent processes. NET-associated histones induce further platelet activation and contribute to vascular damage due to their cytotoxicity.

We determined nucleosomes and citrullinated histone H3 as markers of NET formation. Both were elevated in COVID-19 positive and COVID-19 negative sepsis patients, supporting previous findings by Traby and co-workers, who reported elevated levels of citrullinated histone H3, D-dimer, CRP, IL-6, as well as an increased neutrophil-to-lymphocyte ratio in COVID-19 patients [60].

Extracellular vesicles are increasingly considered as amplifiers of immunothrombosis, particularly the subset of EVs exposing phosphatidylserine on their surface, which propagates thrombin generation [23,24]. In line with previous studies [60,66], we observed elevated levels of phosphatidylserine-exposing EVs in COVID-19 negative as well as COVID-19 positive sepsis patients, the majority of which were platelet-derived. While the levels of platelet-derived EVs did not differ between COVID-19 negative and COVID-19 positive sepsis patients, the concentration of CRP^+^ EVs was higher in COVID-19 positive sepsis patients. The association of CRP with EVs may further support inflammation, as there is increasing evidence that functionally inert pentameric CRP dissociates to pro-inflammatory monomeric CRP upon binding to phosphocholine-enriched membranes [67].

## 5. Conclusions

Our study provides evidence that the profiles of inflammatory mediators largely overlap between COVID-19 negative and COVID-19 positive sepsis patients. Our data support the notion that activated platelets and platelet-derived EVs are potential mediators of immunothrombosis in sepsis and COVID-19.

## Figures and Tables

**Table 1:** Patient characteristics at baseline.

Characteristics	Controls (*n* = 25)	Sepsis − COVID-19 (*n* = 64)	Sepsis + COVID-19 (*n* = 14)
Age, years	34.9 ± 11.7	63.6 ± 18.7	68.8 ± 17.3
Male	10 (40%)	41 (64%)	11 (79%)
Female	15 (60%)	23 (36%)	3 (21%)
ICU admission	n.a.	14 (22%)	5 (36%)
Mortality	n.a.	7 (11%)	6 (43%)
Positive blood culture	n.a.	8 (13%)	1 (7%)
CRP [mg/dL]	n.a.	9.78 (6.31; 12.08)	11.31 (5.67; 15.11)
PCT [µg/L]	n.a.	0.54 (0.31; 3.18)	0.56 (0.16; 1.14)
Red blood cells [× 10^12^/L]	n.a.	3.42 (3.16; 3.57)	3.45 (3.11; 3.97)
White blood cells [× 10^9^/L]	n.a.	9.05 (7.55; 11.2)	8.35 (6.15; 13.65)
Monocytes [× 10^9^/L]	n.a.	0.69 (0.48; 0.83)	0.38 (0.22; 0.64)
Neutrophils [× 10^9^/L]	n.a.	9.4 (4.85; 11)	9.85 (4.85; 13.6)
Lymphocytes [× 10^9^/L]	n.a.	1.03 (0.69; 1.16)	0.98 (0.36; 1.62)
Neutrophil-to-lymphocyte ratio (NLR)	n.a.	7.16 (5.53; 11.25)	16.32 (1.74; 36.36)
Platelets [× 10^9^/L]	257 (222; 303) ^a^	212 (166; 232)	170 (132; 274)
**Comorbidities, *n* (%)**			
Infectious/parasitic diseases	n.a.	19 (30)	4 (29)
Neoplasms	n.a.	4 (6)	5 (36)
Blood/blood forming organ diseases	n.a.	9 (14)	3 (21)
Immune system diseases	n.a.	3 (5)	0 (0)
Endocrine/nutritional/metabolic diseases	n.a.	24 (38)	6 (43)
Mental/behavioral/neurodevelopmental disorders	n.a.	9 (14)	2 (14)
Nervous system diseases	n.a.	3 (5)	4 (29)
Visual system diseases	n.a.	1 (2)	1 (7)
Circulatory system diseases	n.a.	34 (53)	10 (71)
Respiratory system diseases	n.a.	25 (39)	13 (93)
Digestive system diseases	n.a.	11 (17)	5 (36)
Skin diseases	n.a.	4 (6)	1 (7)
Musculoskeletal/connective tissue diseases	n.a.	5 (8)	0 (0)
Genitourinary system diseases	n.a.	29 (45)	6 (43)
Other symptoms, signs, or clinical findings	n.a.	4 (6)	6 (43)
Injury/poisoning/consequences of external causes	n.a.	5 (8)	1 (7)
Factors influencing health status/contact with health services	n.a.	10 (16)	2 (14)

Data are represented as mean ± standard deviation and counts (percentage). Laboratory parameters are reported as median (lower; upper confidence interval). ^a^ *n* = 10; n.a., not applicable; ICU, intensive care unit; CRP, C-reactive protein; PCT, procalcitonin.

**Table 2:** Mediator profiles for controls, sepsis patients, and sepsis patients diagnosed with COVID-19.

Parameter		Controls (*n* = 25)	Sepsis − COVID-19 (*n* = 64)	Sepsis + COVID-19 (*n* = 14)
PF4	[ng/mL]	558 (394; 587)	820 (688; 1330)	1260 (544; 2657)
D-dimer	[ng/mL]	227 (189; 253)	471 (380; 617)	378 (278; 485)
nucleosomes	[AU]	0.01 (0.01; 0.02)	0.09 (0.05; 0.11)	0.11 (0.04; 0.15)
cit H3	[ng/mL]	1 (0.78; 1.32)	2.14 (1.62; 3.06)	2.27 (1.32; 3.04)
HMGB-1	[ng/mL]	1.01 (0.35; 1.25)	3.22 (2.63; 5.69)	7.16 (3.74; 8.01)
C5b-9	[µg/mL]	0.68 (0.63; 0.76)	1.36 (1.2; 1.44)	1.52 (1.29; 1.74)
EVs	[counts/µL]	53,500 (45,000; 66,000)	132,250 (100,250; 188,500)	311,000 (151,000; 630,000)
CD41^+^ EVs	[counts/µL]	9000 (5940; 10,440)	28,945 (23,090; 57,195)	73,085 (39,790; 198,860)
CD41^+^ EVs	[% of total EVs]	15 (11; 18)	27 (18; 34)	31 (23; 35.5)
CRP^+^ EVs	[counts/µL]	2000 (1500; 4000)	71,660 (52,500; 88,500)	106750 (85,000; 263,500)
CRP^+^ EVs	[% of total EVs]	4 (2; 4)	55 (46; 61)	62 (40; 67.5)
IL-1β	[pg/mL]	0.92 (0.72; 1.32)	1.88 (1.28; 1.88)	2.16 (0.96; 2.84)
IL-1ra	[pg/mL]	251 (164; 379)	1010 (431; 1353)	1495 (866; 2805)
IL-2	[pg/mL]	1.7 (n.a.; n.a.)	1.93 (1.7; 2.16)	1.7 (1.7; 1.73)
IL-4	[pg/mL]	3.36 (2.6; 3.56)	3.02 (2.56; 3.58)	3.18 (2.08; 3.84)
IL-5	[pg/mL]	5.34 (n.a.; n.a.)	5.34 (n.a.; n.a.)	5.34 (n.a.; n.a.)
IL-6	[pg/mL]	0.42 (n.a.; n.a.)	28.6 (20.12; 50.04)	34.34 (12.32; 115)
IL-7	[pg/mL]	2.6 (n.a.; n.a.)	10.86 (5.36; 12.64)	12.64 (2.6; 17.68)
IL-8	[pg/mL]	2.76 (1.56; 4.64)	15.44 (13.56; 21.84)	15 (7.68; 22.3)
IL-9	[pg/mL]	557 (493; 591)	383 (353; 402)	402 (303; 440)
IL-10	[pg/mL]	0.81 (n.a.; n.a.)	2.84 (1.84; 3.48)	4.28 (0.81; 9.12)
IL-12	[pg/mL]	1.87 (n.a.; n.a.)	1.87 (n.a.; n.a.)	1.87 (1.83; 2.96)
IL-13	[pg/mL]	0.96 (0.44; 1.2)	2.08 (1.32; 2.56)	3.72 (2.76; 4.26)
IL-15	[pg/mL]	18.02 (n.a.; n.a.)	18.02 (n.a.; n.a.)	18.02 (n.a.; n.a.)
IL-17	[pg/mL]	11.24 (7.32; 11.48)	2.67 (n.a.; n.a.)	2.67 (2.67; 2.67)
IFN-γ	[pg/mL]	4.8 (3.48; 6.04)	5.28 (3.84; 5.62)	6.48 (5.58; 8.34)
TNF-α	[pg/mL]	32.72 (30.56; 34.84)	82.92 (74.44; 93.96)	52.38 (48.24; 85.52)
eotaxin	[pg/mL]	42.12 (30.32; 49.12)	44.48 (36; 53.92)	56.58 (45.76; 61.08)
IP-10	[pg/mL]	297 (202; 354)	846 (614; 1400)	5806 (2593; 8493)
MCP-1	[pg/mL]	23.2 (16.4; 25.52)	34.22 (24.12; 44.64)	98.24 (41.52; 184)
MIP-1α	[pg/mL]	1.28 (1.01; 1.28)	5.42 (4.24; 6.6)	3.2 (2.08; 7.42)
MIP-1β	[pg/mL]	248 (233; 271)	207 (188; 220)	212 (177; 251)
RANTES	[pg/mL]	7742 (4249; 9806)	3940 (2989; 5182)	5319 (2357; 8911)
FGF	[pg/mL]	21.2 (15.4; 22.04)	26.24 (16.16; 27.12)	30.66 (26.48; 33.28)
G-CSF	[pg/mL]	45.68 (31.6; 57.56)	174 (148; 237)	213 (90; 340)
GM-CSF	[pg/mL]	0.44 (n.a.; n.a.)	0.44 (n.a.; n.a.)	0.44 (n.a.; n.a.)
PDGF-bb	[pg/mL]	258 (164; 330)	343 (222; 461)	573 (329; 996)
VEGF	[pg/mL]	4.22 (n.a.; n.a.)	4.22 (n.a.; n.a.)	4.22 (n.a.; n.a.)

Data are represented as median (lower; upper confidence interval). n.a., not applicable as confidence intervals could not be calculated due to low variations.

## Data Availability

All authors confirm that all relevant data are included in the article. Additional statistical data are available from the corresponding author upon request.

## Supplementary Materials

The following supporting information can be downloaded at: https://www.mdpi.com/article/10.3390/diagnostics13061069/s1, Figure S1: Calibration and controls for the flow cytometric characterization of extracellular vesicles.; Figure S2: Spearman-based correlation plot of all inflammatory mediators analyzed in COVID-19 negative sepsis patients.; Figure S3: Spearman-based correlation plot of all inflammatory mediators analyzed in COVID-19 positive sepsis patients.; Table S1: Antibodies and fluorochrome conjugates used for flow cytometric characterization of extracellular vesicles.; Table S2: Mediator profiles in sepsis patients with unknown and confirmed bacteremia.; Table S3: Inflammatory mediator profiles in survivors and non-survivors.
