# Supplementary material for: Infection with SARS-CoV-2 Is Associated with Elevated Levels of IP-10, MCP-1, and IL-13 in Sepsis Patients
Source: Diagnostics (Basel). 2023 Mar 11;13(6):1069. doi: 10.3390/diagnostics13061069 (PMC10047668; doi:10.3390/diagnostics13061069)
Supplement: Supplementary file 1 [file diagnostics-13-01069-s001.zip › diagnostics-2266090-supplementary.pdf]

Supplementary Table S1. Antibodies and fluorochrome conjugates used for flow cytometric characterization of extracellular vesicles.

Flow cytometry						
**Antigen**	**Origin**	**Clone**	**Marker for**	**Fluorochrome**	**Abbreviation**	**Supplier**
CD41	mouse	P2	platelets	Phycoerythrin Cyanin 7	PC7	Beckman Coulter
PF4	mouse	# 170138	platelet factor 4	Phycoerythrin	PE	R&D Systems
CRP	goat	-	C-reactive protein	Fluorescein Isothiocyanate	FITC	Abcam
Anx5	-	-	phosphatidyl-serine	Allophyco-Cyanin	APC	BD Biosciences

Supplementary Table S2. Mediator profiles in sepsis patients with unknown and confirmed bacteremia.

**Parameter**		**Blood Culture negative (*n*=69)**	**Blood Culture positive (*n*=9)**
CRP	[mg/dL]	8.96 (5.67; 11.51)	12.94 (9.92; 20.27)
PCT	[μg/L]	0.42 (0.2; 0.54)	16.78 (1.61; 71.46)
red blood cells	[x 10^{12}/L]	3.44 (3.24; 3.58)	3.02 (2.71; 3.71)
white blood cells	[x 10^9/L]	8.6 (7.4; 10)	14.4 (4.6; 18.2)
monocytes	[x 10^9/L]	0.63 (0.38; 0.73)	0.57 (0.06; 1.05)
neutrophils	[x 10^9/L]	8.6 (5.7; 10.7)	13.6 (3.2; 14.4)
lymphocytes	[x 10^9/L]	1.03 (0.8; 1.19)	0.51 (0.18; 1.15)
NLR	-	7.33 (5.55; 11.88)	13.13 (4.71; 28.96)
platelets	[x 10^9/L]	207 (163; 221)	173 (35; 300)
PF4	[ng/mL]	955 (740; 1368)	344 (94.5; 2698)
D-dimer	[ng/mL]	396 (368; 516)	840 (471; 1492)
nucleosomes	[AU]	0.09 (0.06; 0.12)	0.09 (0.03; 0.21)
cit H3	[ng/mL]	2.07 (1.35; 2.67)	2.62 (2.13; 3.72)
HMGB-1	[ng/mL]	4.34 (2.86; 7.16)	5.09 (2.63; 9.91)
C5b-9	[μg/mL]	1.38 (1.29; 1.45)	1.55 (0.77; 1.73)
EVs	[counts/μL]	176,500 (120,000; 234,500)	129,000 (98,500; 176,500)
$CD41^+$ EVs	[counts/μL]	48,100 (25,125; 64,405)	39,790 (12,240; 74,130)
$CD41^+$ EVs	[% of total EVs]	27 (18; 32)	34 (12; 42)
CRP^+ EVs	[counts/μL]	83,500 (61,000; 101,500)	77,000 (46,000; 112,960)
CRP^+ EVs	[% of total EVs]	55 (46; 61)	60 (46; 62)
IL-1β	[pg/mL]	1.88 (1.56; 2.46)	2.08 (0.27; 4.44)
IL-1ra	[pg/mL]	1011 (431; 1285)	1552 (647; 16296)
IL-2	[pg/mL]	1.7 (n.a.; n.a.)	2.19 (1.7; 8.84)
IL-4	[pg/mL]	3.02 (2.56; 3.44)	3.56 (1.6; 5.4)
IL-5	[pg/mL]	5.34 (n.a.; n.a.)	23.65 (5.34; 257)
IL-6	[pg/mL]	26.52 (16.5; 50.04)	35.98 (27.64; 1752)
IL-7	[pg/mL]	11.2 (4.64; 12.64)	8.74 (2.6; 12.64)
IL-8	[pg/mL]	15.44 (12.96; 20.52)	20.74 (12.6; 160)
IL-9	[pg/mL]	386 (358; 402)	408 (124; 467)
IL-10	[pg/mL]	2.84 (1.32; 2.92)	2.5 (0.81; 65.84)
IL-12	[pg/mL]	1.87 (n.a.; n.a.)	2.96 (0.81; 12.24)
IL-13	[pg/mL]	2.42 (2.08; 2.76)	1.16 (0.36; 5.4)
IL-15	[pg/mL]	18.02 (n.a.; n.a.)	18.02 (n.a.; n.a.)
IL-17	[pg/mL]	2.67 (n.a.; n.a.)	10.96 (2.67; 28.48)
IFN-γ	[pg/mL]	5.32 (4.68; 6.2)	11.9 (6.12; 54.4)
TNF-α	[pg/mL]	74.76 (66.24; 84.64)	108 (86.88; 233)
eotaxin	[pg/mL]	48.66 (39.48; 55.44)	49.26 (24.24; 72)
IP-10	[pg/mL]	1344 (817; 2434)	690 (614; 7106)
MCP-1	[pg/mL]	37.72 (27.34; 46.92)	42.3 (22.36; 448)
MIP-1α	[pg/mL]	4.52 (3.52; 6.24)	10.42 (5.08; 26.92)
MIP-1β	[pg/mL]	205 (189; 219)	224 (67.96; 347)
RANTES	[pg/mL]	4360 (3135; 5281)	2778 (529; 6211)
FGF	[pg/mL]	26.48 (20; 29.28)	30.82 (26.24; 62.44)
G-CSF	[pg/mL]	165 (123; 216)	376 (214; 1492)
GM-CSF	[pg/mL]	0.44 (n.a.; n.a.)	0.44 (n.a.; n.a.)
PDGF-bb	[pg/mL]	391 (248; 554)	204 (2.46; 621)
VEGF	[pg/mL]	4.22 (n.a.; n.a.)	4.22 (n.a.; n.a.)

Data are represented as median (lower; upper confidence interval). n.a., not applicable as confidence intervals could not be calculated due to low variations.

Supplementary Table S3. Inflammatory mediator profiles in survivors and non-survivors.

Parameter		Survivors	Non-Survivors
CRP	[mg/dL]	9.7 (5.98; 12.08)	9.92 (5.67; 16.19)
PCT	[μg/L]	0.44 (0.22; 0.75)	2.06 (0.48; 12.32)
red blood cells	[x 10^{12}/L]	3.5 (3.22; 3.61)	3.29 (2.85; 3.36)
white blood cells	[x 10^{9}/L]	9 (7.5; 12.1)	8.6 (4.4; 9.7)
monocytes	[x 10^{9}/L]	0.68 (0.53; 0.76)	0.36 (0.22; 0.72)
neutrophils	[x 10^{9}/L]	10.25 (5.7; 11.2)	8 (3.75; 12.5)
lymphocytes	[x 10^{9}/L]	1.02 (0.72; 1.16)	1.02 (0.4; 1.39)
NLR	-	7.33 (5.57; 13.15)	11.87 (0.99; 31.96)
platelets	[x 10^{9}/L]	211 (163; 232)	168 (27; 212)
PF4	[ng/mL]	901 (732; 1463)	791 (260; 1618)
D-dimer	[ng/mL]	459 (373; 596)	401 (278; 541)
nucleosomes	[AU]	0.09 (0.05; 0.11)	0.11 (0.05; 0.2)
cit H3	[ng/mL]	2.1 (1.6; 2.73)	2.64 (1.32; 3.39)
HMGB-1	[ng/mL]	3.86 (2.86; 7.27)	4.58 (2.1; 10.17)
C5b-9	[μg/mL]	1.35 (1.25; 1.45)	1.47 (1.09; 1.63)
EVs	[counts/μL]	185,000 (117,500; 236,500)	126,500 (82,000; 148,000)
$CD41^{+}$ EVs	[counts/μL]	48,100 (25,125; 69,625)	31,980 (11,880; 54,360)
$CD41^{+}$ EVs	[% of total EVs]	28 (21; 34)	23 (17; 34)
CRP^{+} EVs	[counts/μL]	85,000 (61,000; 104,500)	79,500 (48,000; 88,500)
CRP^{+} EVs	[% of total EVs]	55 (46; 60)	63 (45; 67)
IL-1β	[pg/mL]	1.88 (1.28; 1.88)	2.66 (0.84; 3.16)
IL-1ra	[pg/mL]	1044 (623; 1353)	1449 (10.76; 4599)
IL-2	[pg/mL]	1.7 (n.a.; n.a.)	1.73 (1.7; 6.68)
IL-4	[pg/mL]	3.08 (2.48; 3.28)	2.48 (1.84; 2.96)
IL-5	[pg/mL]	5.34 (n.a.; n.a.)	5.34 (n.a.; n.a.)
IL-6	[pg/mL]	27.16 (17.68; 38.68)	66.62 (23.66; 126)
IL-7	[pg/mL]	11.2 (4.64; 12.64)	6.72 (4.64; 15.46)
IL-8	[pg/mL]	15.44 (13.56; 21.84)	21.18 (13.06; 34.22)
IL-9	[pg/mL]	387 (360; 413)	370 (288; 423)
IL-10	[pg/mL]	2.62 (1.58; 3.68)	3.96 (1.84; 7.24)
IL-12	[pg/mL]	1.87 (n.a.; n.a.)	2.09 (1.87; 2.96)
IL-13	[pg/mL]	2.4 (1.32; 2.76)	2.58 (2.08; 4.28)
IL-15	[pg/mL]	18.02 (n.a.; n.a.)	18.02 (n.a.; n.a.)
IL-17	[pg/mL]	2.67 (n.a.; n.a.)	2.67 (2.67; 10.16)
IFN-γ	[pg/mL]	5.88 (4.6; 6.2)	5.62 (4.84; 10.52)
TNF-α	[pg/mL]	78.4 (68.44; 92.48)	93.62 (52.76; 119)
eotaxin	[pg/mL]	47.2 (38.4; 55.44)	49.38 (25.4; 60.18)
IP-10	[pg/mL]	978 (658; 1721)	5639 (655; 9819)
MCP-1	[pg/mL]	35.06 (26.12; 46.4)	74.2 (27.48; 114)
MIP-1α	[pg/mL]	4.88 (3.64; 6.36)	6.34 (3.08; 30.12)
MIP-1β	[pg/mL]	209 (188; 221)	197 (165; 256)
RANTES	[pg/mL]	4527 (3081; 5281)	3117 (1586; 9006)
FGF	[pg/mL]	26.48 (20; 29.68)	27.88 (9.44; 29.32)
G-CSF	[pg/mL]	192 (134; 237)	192 (128; 893)
GM-CSF	[pg/mL]	0.44 (n.a.; n.a.)	0.44 (n.a.; n.a.)
PDGF-bb	[pg/mL]	401 (254; 565)	237 (35.32; 688)
VEGF	[pg/mL]	4.22 (n.a.; n.a.)	4.22 (n.a.; n.a.)

Data are represented as median (lower; upper confidence interval). n.a., not applicable as confidence intervals could not be calculated due to low variations.

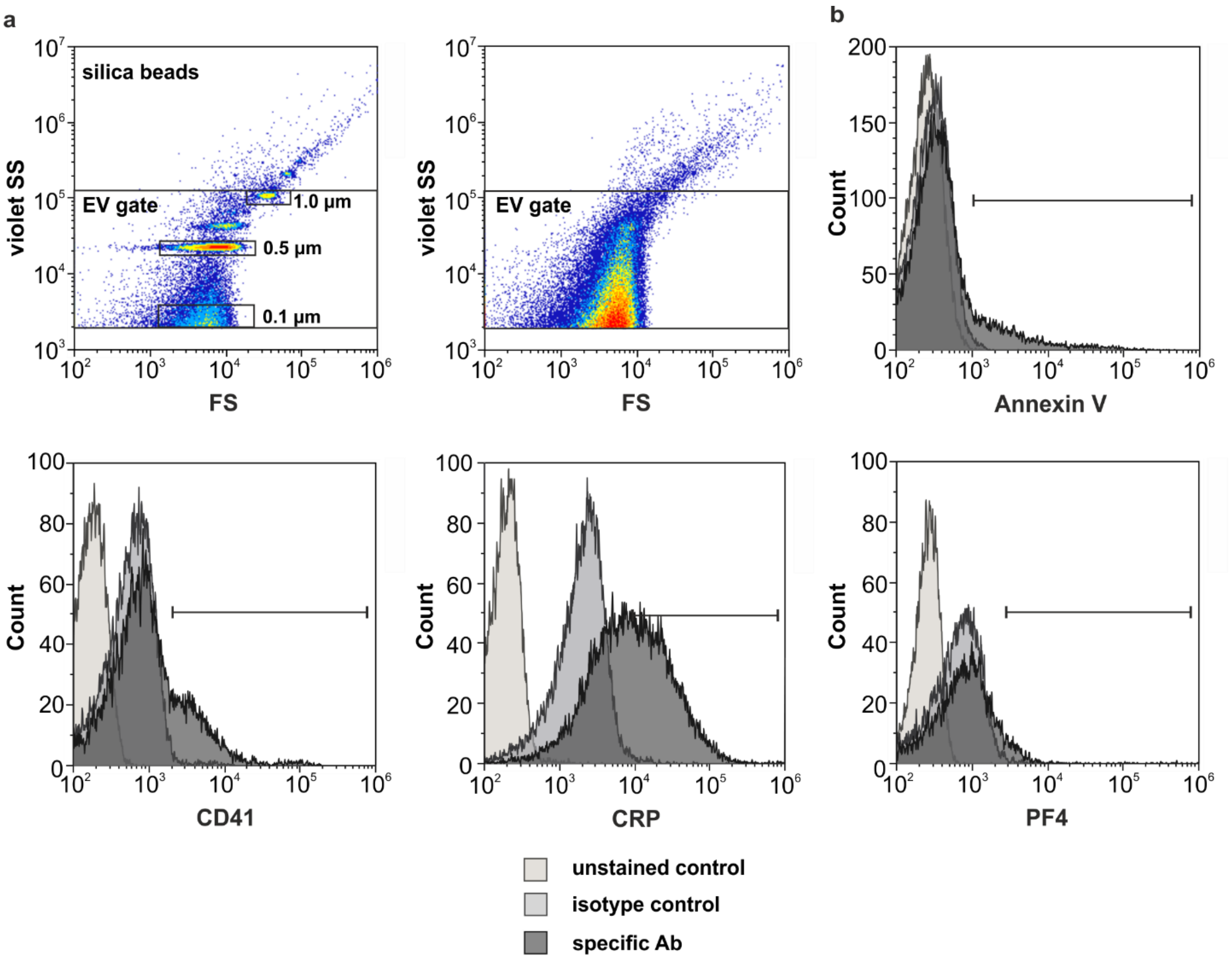

Supplementary Figure S1. Calibration and controls for the flow cytometric characterization of extracellular vesicles. (a) Flow cytometric characterization was performed on a CytoFLEX LX flow cytometer (Beckman Coulter) after calibration with fluorescent silica particles (0.1, 0.5, and 1.0 µm), and the EV gate was set below the 1 µm bead cloud as described in the Methods section (left panel). Staining of EVs was performed as described in the methods section and a representative forward scatter *vs.* violet side scatter (FS *vs.* violet SS) density plot (right panel) is shown. (b) The respective unstained controls, isotype controls, and single stainings are shown. Annexin V staining in PBS without Ca^{++} and Mg^{++} was used as negative control. Bars indicate positive expression.

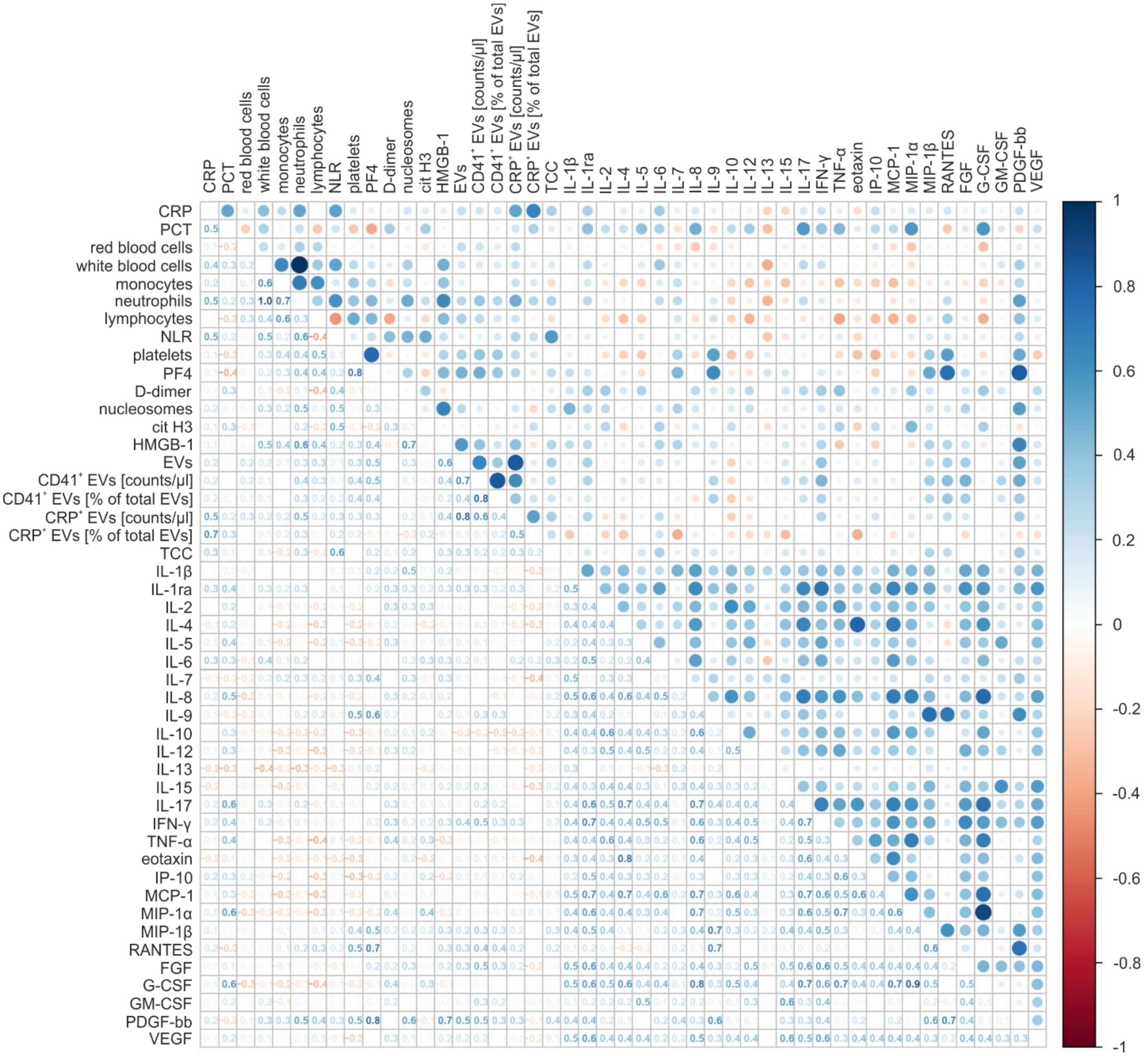

Supplementary Figure S2. Spearman-based correlation plot of all inflammatory mediators analyzed in COVID-19 negative sepsis patients. The colour and size of the dots represent the level of correlation coefficient.

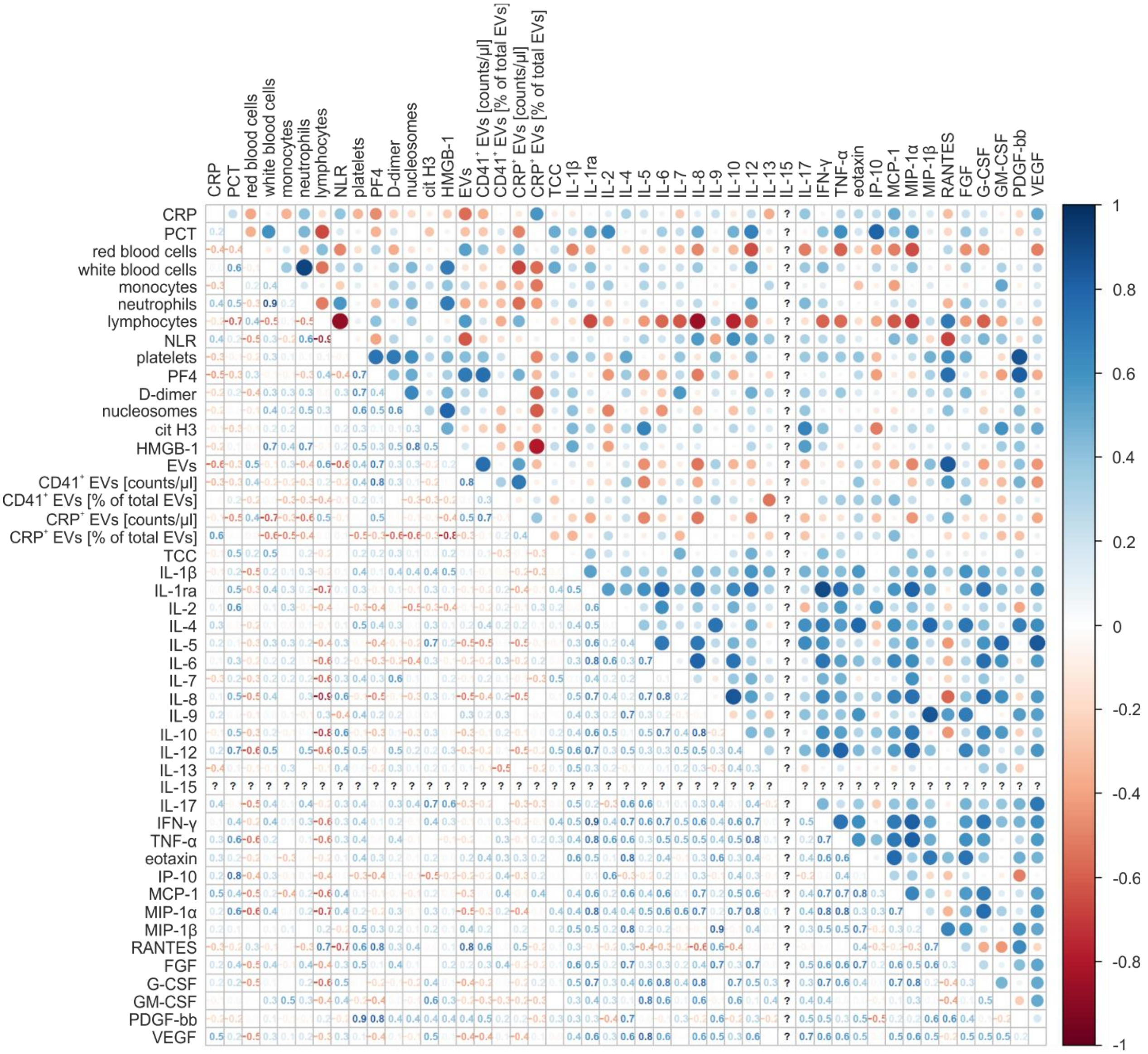

Supplementary Figure S3. Spearman-based correlation plot of all inflammatory mediators analyzed in COVID-19 positive sepsis patients. The colour and size of the dots represent the level of correlation coefficient. For IL-15 correlations could not be calculated due to low variations.
